# Supplementary material for: Dynamics of anti-Strongyloides IgG antibody responses and implications for strongyloidiasis surveillance in rural Amazonians: A population-based panel data analysis
Source: PLoS Negl Trop Dis. 2025 Apr 1;19(4):e0012967. doi: 10.1371/journal.pntd.0012967 (PMC11978073; doi:10.1371/journal.pntd.0012967)
Supplement: S1 Fig — The map also shows the village of Nova Califórnia (western Rondônia State), the nearest town, Acrelândia (eastern Acre State), where our field laboratory (where blood and stool samples were processed) is situated, and the BR 364 interstate highway, which connects Acre, Rondônia and southern Amazonas to the rest of the country. Source [17]:. The source is an open-access article distributed under the terms of the Creative Commons Attribution License, which permits unrestricted use, distribution, and reproduction in any medium, provided the original author and source are credited. (PDF) [file pntd.0012967.s002.pdf]

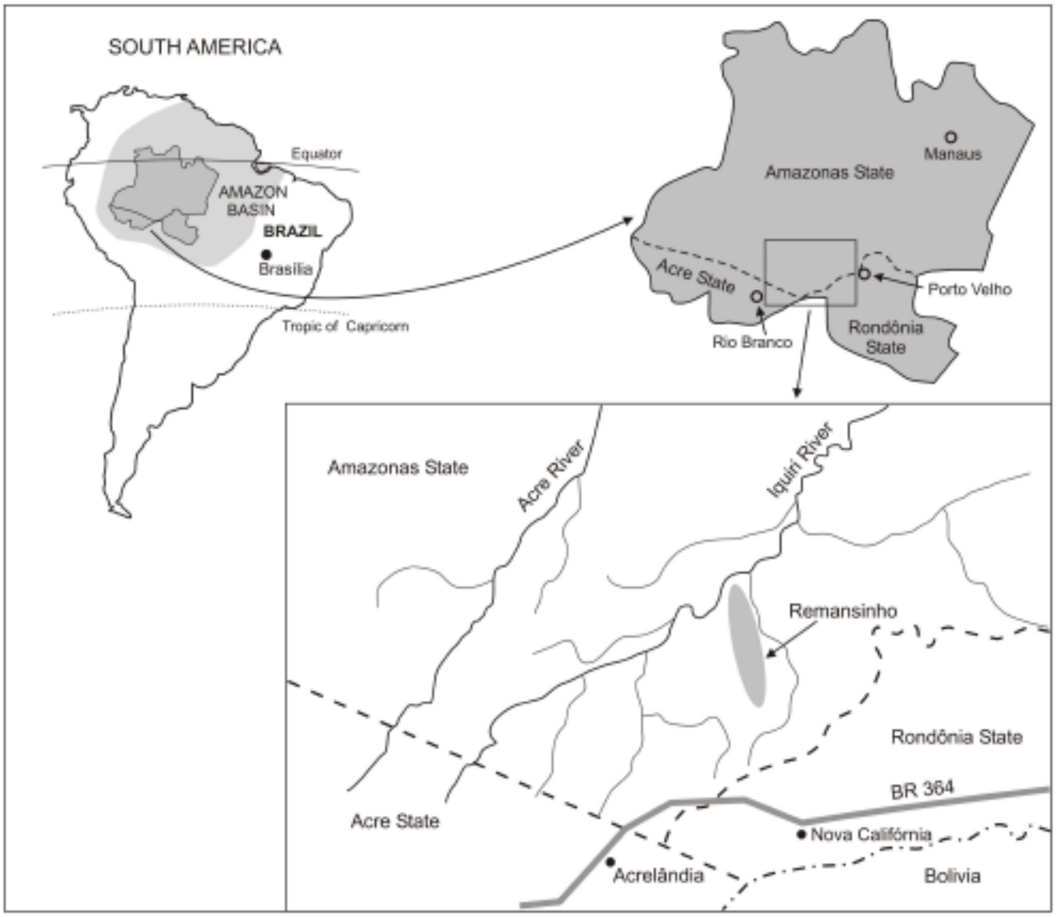

S1 Figure. Location of the field site, Remansinho, southern Amazonas State, Brazilian Amazonia. The map also shows the village of Nova Califórnia (western Rondônia State), the nearest town, Acrelândia (eastern Acre State), where our field laboratory (where blood and stool samples were processed) is situated, and the BR 364 interstate highway, which connects Acre, Rondônia and southern Amazonas to the rest of the country. Source: Barbosa et al. (2014) [17]. The source is an open-access article distributed under the terms of the Creative Commons Attribution License, which permits unrestricted use, distribution, and reproduction in any medium, provided the original author and source are credited.
