# Supplementary material for: Dynamics of anti-Strongyloides IgG antibody responses and implications for strongyloidiasis surveillance in rural Amazonians: A population-based panel data analysis
Source: PLoS Negl Trop Dis. 2025 Apr 1;19(4):e0012967. doi: 10.1371/journal.pntd.0012967 (PMC11978073; doi:10.1371/journal.pntd.0012967)
Supplement: S1 Table — (PDF) [file pntd.0012967.s003.pdf]

S1 Table. Proportion of stool samples positive for intestinal parasites at baseline in the population of five farming settlements of Amazonas State, Brazil, 2010

	Nr. positive, N (%)	Nr. IgG-positive, n/N
**Helminths**		
Ascaris lumbricoides	4 (2.6%)	1/4
Strongyloides stercoralis	2 (1.3%)	2/2
Trichuris trichiura	5 (3.3%)	3/5
Hymenolepis nana	2 (1.3%)	1/2
Hookworm	4 (2.6%)	2/4
Any helminth	14 (9.2%)	7/14
**Protozoa**		
Entamoeba coli	8 (5.3%)	4/8
Giardia duodenalis	4 (2.6%)	1/4
Iodoamoeba butschlii	1 (0.7%)	1/1
Entamoeba histolytica	1 (0.7%)	0/1
Endolimax nana	3 (2.0%)	0/3

A total of 152 formalin-preserved stool samples (one per participant) were tested for cysts, ova, and larvae by microscopy using a standard sedimentation-concentration technique in test tubes. Some patients were infected with two or more parasite species. Both participants infected with *Strongyloides stercoralis* (one single-species infection and one mixed-species infection) were prescribed ivermectin (200 µg/kg/day orally for 2 days), but the treatment was not supervised. One of them (coinfected with *Trichuris trichiura*) remained seropositive during the subsequent surveys, with high absorbance values (above the overall median of 0.561) measured in plasma samples tested in 2011, 2012, and 2013. The other participant with microscopy-confirmed infection was lost for follow-up.
