# Supplementary material for: Dynamics of anti-Strongyloides IgG antibody responses and implications for strongyloidiasis surveillance in rural Amazonians: A population-based panel data analysis
Source: PLoS Negl Trop Dis. 2025 Apr 1;19(4):e0012967. doi: 10.1371/journal.pntd.0012967 (PMC11978073; doi:10.1371/journal.pntd.0012967)
Supplement: S3 Table — (PDF) [file pntd.0012967.s005.pdf]

S3 Table. Pairwise comparisons of anti-*Strongyloides* IgG status (stratified as negative, low, and high) in consecutive surveys of the population of five farming settlements in Amazonas State, Brazil, with blood draws approximately 24 months apart (2010 vs. 2012, 2011 vs. 2013, and 2012 vs. 2014).

	**IgG status in 2012**			
**IgG status in 2010**	**Negative**	**Low**	**High**	**Total**
**Negative**	34 (56.7%)	24 (40.0%)	3 (3.3%)	60
**Low**	7 (38.9%)	7 (38.9%)	4 (22.2%)	18
**High**	1 (5.3%)	3 (15.8%)	15 (78.9%)	19
**Total**	42	34	21	97
	**IgG status in 2013**			
**IgG status in 2011**	**Negative**	**Low**	**High**	**Total**
**Negative**	28 (70.0%)	11 (27.5%)	1 (2.5%)	40
**Low**	8 (32.0%)	8 (32.0%)	9 (36.0%)	25
**High**	0 (0.0%)	1 (4.3%)	22 (95.6%)	23
**Total**	36	20	32	88
	**IgG status in 2014**			
**IgG status in 2012**	**Negative**	**Low**	**High**	**Total**
**Negative**	30 (78.9%)	6 (15.8%)	2 (5.3%)	38
**Low**	4 (21.0%)	13 (68.4%)	2 (10.5%)	19
**High**	0 (0.0%)	5 (25.0%)	15 (75.0%)	20
**Total**	34	24	19	77

IgG responses to *Strongyloides* were stratified as negative (absorbance ≤ 0.286), low (absorbance between 0.287 and 0.561) and high (absorbance > 0.561), with 0.561 corresponding to the median absorbance value among positive samples during the study. Percentages were calculated within rows. Percentages were calculated within rows. Data from this table were combined to prepare Figure 1B (see the main text).
