# Supplementary material for: Dynamics of anti-Strongyloides IgG antibody responses and implications for strongyloidiasis surveillance in rural Amazonians: A population-based panel data analysis
Source: PLoS Negl Trop Dis. 2025 Apr 1;19(4):e0012967. doi: 10.1371/journal.pntd.0012967 (PMC11978073; doi:10.1371/journal.pntd.0012967)
Supplement: S6 Table — (PDF) [file pntd.0012967.s008.pdf]

S6 Table. Median levels of anti-*Strongyloides* IgG levels (absorbance values) in relation to the time of the seroconversion event (between 2010 and 2013) in the population of five farming settlements in Amazonas State, Brazil.

	**Levels of anti-*Strongyloides* IgG antibodies**		
**Months in relation to seroconversion**	**Median**	**Interquartile range**	**Number tested**
36 months before	0.313	0.213-0.450	8
24 months before	0.223	0.192-0.272	28
12 months before	0.233	0.191-0.260	53
At seroconversion	0.355	0.326-0.428	53
After 12 months	0.315	0.244-0.463	23
After 24 months	0.340	0.237-0.550	11
After 36 months	0.395	0.377-0.434	5

Medians for each time interval calculated by combining IgG antibody measurements for 53 seroconversion events in 51 study participants (see Figure 2).
