# Supplementary material for: Dynamics of anti-Strongyloides IgG antibody responses and implications for strongyloidiasis surveillance in rural Amazonians: A population-based panel data analysis
Source: PLoS Negl Trop Dis. 2025 Apr 1;19(4):e0012967. doi: 10.1371/journal.pntd.0012967 (PMC11978073; doi:10.1371/journal.pntd.0012967)
Supplement: S7 Table — (PDF) [file pntd.0012967.s009.pdf]

S7 Table. Spearman correlation test results for anti-*Strongyloides* IgG levels (absorbance values) measured in consecutive surveys (between 2010 and 2014) of the population of five farming settlements in Amazonas State, Brazil.

Year of survey	2010	2011	2012	2013	2014
**2010**		0.797	0.636	0.722	0.742
**2011**	125		0.726	0.801	0.806
**2012**	97	141		0.819	0.756
**2013**	71	88	95		0.850
**2014**	45	59	77	47	

Above the diagonal: Spearman's correlation test results (r_s) for each pairwise comparison; $P < 0.0001$ for all. Below the diagonal: numbers of sample pairs in each comparison.
