# Supplementary material for: Dynamics of anti-Strongyloides IgG antibody responses and implications for strongyloidiasis surveillance in rural Amazonians: A population-based panel data analysis
Source: PLoS Negl Trop Dis. 2025 Apr 1;19(4):e0012967. doi: 10.1371/journal.pntd.0012967 (PMC11978073; doi:10.1371/journal.pntd.0012967)
Supplement: Attachment — Submitted filename: Rebuttal Letter.docx [file pntd.0012967.s013.docx]

Rebuttal Letter: PNTD-D-24-01554 – “Dynamics of anti-*Strongyloides* IgG antibody responses and implications for strongyloidiasis surveillance in rural Amazonians: a population-based panel data analysis”

Dear Editors,

We sincerely thank both reviewers and the associate editors for their insightful comments and suggestions. Here, we submit a carefully revised version of the manuscript that addresses all comments and suggestions raised during the review process. We hope that this extensively revised version of the manuscript is much clearer and more attractive for the broad audience of PLoS NTD.

**1. Editor Comments:**

Dear authors, the reviewers of this article have pointed out some issues that need to be addressed, especially in relation to methodology. Please address all comments, even if the authors disagree with any of the questions or suggestions.

Thanks for the careful and extensive review. Here, we provide point-by-point responses to all comments and suggestions.

**2. Journal Requirements:**

1) Tables should not be uploaded as individual files. Please remove these files and include the Tables in your manuscript file as editable, cell-based objects.

Done as requested.

2) Figure S1. Please provide a direct link to the base layer of the map (i.e., the country or region border shape) and ensure this is also included in the figure legend; and provide a link to the terms of use / license information for the base layer image or shapefile. We cannot publish proprietary or copyrighted maps (e.g. Google Maps, Mapquest) and the terms of use for your map base layer must be compatible with our CC BY 4.0 license.

Note: if you created the map in a software program like R or ArcGIS, please locate and indicate the source of the basemap shapefile onto which data has been plotted.

If your map was obtained from a copyrighted source please amend the figure so that the base map used is from an openly available source. Alternatively, please provide explicit written permission from the copyright holder granting you the right to publish the material under our CC BY 4.0 license.

If you are unsure whether you can use a map or not, please do reach out and we will be able to help you. The following websites are good examples of where you can source open access or public domain maps:

* U.S. Geological Survey (USGS) - All maps are in the public domain. (http://www.usgs.gov)

* PlaniGlobe - All maps are published under a Creative Commons license so please cite u201cPlaniGlobe, http://www.planiglobe.com, CC BY 2.0u201d in the image credit after the caption. (http://www.planiglobe.com/?lang=enl)

* Natural Earth - All maps are public domain. (http://www.naturalearthdata.com/about/terms-of-use/).

Figure S1 was originally published as Figure 1 of Barbosa et al., 2014 (doi:10.1371/journal.pntd.0003109). It has originally been drawn by hand by Mr. Cassiano Pereira, an illustrator formerly affiliated with our Department who passed away a couple of years ago. As far as I know, Mr. Pereira did not use any base layer image or shapefile. I asked the PLoS NTD editorial team whether it would be ok to reproduce this figure as Figure S1 in the present manuscript and just received the following response (February 6^th^, 2025):

“Dear Dr Ferreira,

Thank you for your email. I have noted the copyright related information for your submission. The source of the maps in S1 Fig appears to be acceptable. Please consider this issue as solved.

We look forward to your resubmission.

Kind regards,

Olena Szabo

Journal Assistant, Editorial Office Ltd.

On behalf of PLOS Neglected Tropical Diseases”

3) Please ensure that the funders and grant numbers match between the Financial Disclosure field and the Funding Information tab in your submission form. Note that the funders must be provided in the same order in both places as well. State the initials, alongside each funding source, of each author to receive each grant. For example: "This work was supported by the National Institutes of Health (####### to AM; ###### to CJ) and the National Science Foundation (###### to AM).".

We revised the funding statement, which now reads:

“This research was supported by the National Institute of Allergy and Infectious Diseases, National Institutes of Health, United States of America (NIAID, https://www.niaid.nih.gov/) (U19 AI089681 to MUF), the Fundação de Amparo à Pesquisa do Estado de São Paulo, Brazil (FAPESP, https://fapesp.br/en) (2009/52729-9 to MUF, 2013/04236-9 to RCBG, and 2022/02401-1 to FMP), and the Fundação para a Ciência e Tecnologia, Portugal (FCT; https://www.fct.pt/en/), institutional projects UID/04413/2020 and LA-REAL LA/P/0117/2020 to MUF. FMP, KKGS, and MUF receive senior researcher scholarships from the Conselho Nacional de Desenvolvimento Científico e Tecnológico of Brazil (CNPq, https://www.gov.br/cnpq/pt-br). The funders had no role in study design, data collection and interpretation, or the decision to submit the work for publication.”

**Additional comments:**

1) Thank you for stating: "Written informed consent was obtained from all study participants >18 years of age or their parents or caregivers”. If child participants, the statement must declare that formal consent (must state whether verbal/written) was obtained from the parent/guardian.

Done. The following information was added: “Written informed consent was obtained from all study participants >18 years of age or from parents or caregivers of children. Participants aged 6-18 years also were requested to sign an assent form; those unable to sign their name provided a thumb print.”

Indeed, according to the Brazilian law, children and adolescents aged 6-18 years must sign an **assent form** and their parents or caregivers must sign the **consent form**. Separate consent forms are provided to children (simpler language) and adults.

2) Thank you for stating"Our dataset is publicly available at the ClinEpiDB repository https://clinepidb.org/ce/app/workspace/analyses/DS_8e1f623542/new/details [ Access: Controlled]

Please note that, though access restrictions are acceptable now, your entire minimal dataset will need to be made freely accessible if your manuscript is accepted for publication. This policy applies to all data except where public deposition would breach compliance with the protocol approved by your research ethics board. If you are unable to adhere to our open data policy, please kindly revise your statement to explain your reasoning and we will seek the editor's input on an exemption.

Because the original study was supported by NIAID/NIH, we were requested to deposit our datasets in the ClinEpiDB repository. However, public funding for ClinEpiDB has decreased over the years and the repository now is relatively slow in responding to requests from researchers.

We aim to fully adhere to the PLoS open data policy. Therefore, we decided to provide the original datasets as supplementary material for publication online (S1 and S2 databases). We changed the Data Availability Statement to: “All relevant data are within the paper and its Supporting Information files (S1 and S2 Databases)”.

Meanwhile, we will deposit the datasets in our institutional repository at the University of São Paulo. This will be done by the time the manuscript is accepted for publication. We have already requested authorization for data deposit (https://uspdigital.usp.br/repositorio/minhas-solicitacoes.jsp?codmnu=10067)

3) Please ensure that the funders and grant numbers match between the Financial Disclosure field and the Funding Information tab in your submission form. Note that the funders must be provided in the same order in both places as well.

Currently, the grant number "LA-REAL LA/P/0117/2020" is missing from the Funding Information and also the order is different in both the places.

Corrected as requested.

**3. Reviewers' Comments:**

Methods:

Reviewer #2: The laboratory procedures are well described.

The statistical analysis is mostly well described, but see my specific feedback under general comments with regards to the description of how the logistic regression model was built.

Please refer to our point-to-point responses below (page 13).

Although the ethics statement is thorough, it doesn't mention the original purpose of sampling (presumably for a malaria study) and any relevance of this.

Thanks for your comments.

At the time this project was evaluated by the Institutional Review Board of the University Hospital of the University of São Paulo and subsequently by the National Human Research Ethics Committee of the Ministry of Health of Brazil, in early 2010, there was no National legislation addressing the establishment of biological collections resulting from field-based surveys. Although the original research project focused on acute febrile illnesses, such as malaria and dengue, the research plan explicitly included further screening for other health conditions that were judged clinically or epidemiologically relevant by the field physicians and nurses. Under this criterion, additional testing was done, at discretion of the field team or by request of study participants, for cutaneous leishmaniasis (by microscopy), Mayaro fever (by RT-PCR), intestinal parasites (by stool examination, as described in the present manuscript), and anemia (hemoglobin measurement). We detected anti-*Strongyloides* antibodies on plasma samples from study participants stored in our biological collection at the Institute of Biomedical Sciences, University of São Paulo, because a high seroprevalence of strongyloidiasis has recently been described in a similar Amazonian setting (doi: 10.1590/S1678-9946202466072).

We added the following sentence to the “Ethics statement: “Plasma samples tested for anti-*Strongyloides* antibodies were part of the biological collection, maintained at the Institute of Biomedical Sciences, University of São Paulo, that resulted from 10 consecutive cross-sectional surveys carried out in the study site.”

At the time of the house-to-house surveys, study participants agreed to provide blood samples for malaria-related tests and other health conditions that were judged clinically or epidemiologically relevant by the field physicians and nurses, but strongyloidiasis was not explicitly mentioned in our consent forms.

Results:

Reviewer #2: The analysis presented broadly matches the analysis plan, although I think the order of the results section could be revised (starting with clarification over which samples were tested, and for what - serology, faecal etc, with timings of surveys). As per my specific feedback in the summary and general comments below, the tables and figures need some revision to enhance clarity.

Thanks again. Please see below our responses to specific questions.

Conclusions:

Reviewer #2: Limitations – the samples were collected over ten years ago, this should be discussed.

Conclusion – Do you really mean a diagnostic method for clinical purposes (i.e. treating on the basis of positive serology)? Or do you recommend the method more for epidemiological surveillance and to aid decision making on control programmes?

First point: The reviewer is correct in pointing that the use of relatively old samples for antibody screening is a potential limitation of the present study. We added the following sentence to the last but one paragraph of Discussion:

“An additional limitation is the use of plasma samples collected over one decade ago. Because these samples have been stored at -70°C before testing, a substantial decrease in antibody concentrations since collection is not expected. However, this study describes antibody prevalence rates between 2010 and 2014, with no further follow-up. Further studies are needed to explore the current clinical and public health significance of anti-*Strongyloides* antibody carriage and how antibody status varies over time in this and other endemic settings and in response to mass treatment, and to identify serological correlates of chronic infection.”

Second point: We suggest the use of serology only for epidemiological surveillance. The (revised) last paragraph of Discussion reads:

“In conclusion, our results further support the use of serology as a diagnostic method that can be implemented in the field, especially as a tool for monitoring temporal changes in *S. stercoralis* transmission intensity in remote populations and estimating the effectiveness of control interventions at a population level.”

We note that some attending physicians in Europe suggest that a positive serology for *Strongyloides* can be taken as evidence of current/recent infection among travelers and immigrants, but we do not advocate for the use of serology in clinical settings in the Amazon, where patients are likely to be exposed to other soil-transmitted helminths that may induce cross-reactive antibodies.

Editorial and Data Presentation Modifications?

Reviewer #1: Figure 1 is somewhat confusing, and the authors should find a way to include the numbers of 'converters' into their figure.

We agree that further information would be required to properly interpret data shown in the revised version of Figure 1, which is a standard Sankey diagram (e.g., https://developers.google.com/chart/interactive/docs/gallery/Sankey).

Adding more numbers to Figure 1 would render it too busy. We instead added new rows to Supplementary Table S2 showing the numbers of “converters” and “nonconverters” (italicized numbers in the 26^th^ row) within an interval of 12 months. Note that, out of 213 initially seronegative people, 47 (22.1%) had low antibody titers 12 months later and 6 (2.8%) had high antibody titers 12 months later. Therefore, there were 53 seroconvertion events (i.e., 24.9% of the participants who were initially seronegative had seroconverted 12 months later).

We added the following sentence to the footnote of the revised version of S2 Table: “Italicized numbers indicate the outcomes of initially seronegative participants 12 months later.”

Moreover, we added the following explanation to the main text:

“To estimate the seroconversion rate within one year, we analyzed 213 sample pairs from study participants with an antibody-negative test who were reassessed approximately 12 months later (Fig. 1A), with a total of 216.3 person-years of follow-up. We observed 53 seroconversion events among 51 participants (two participants seroconverted twice): 20 events between 2010 and 2011 (74 sample pairs tested), 18 between 2011 and 2012 (70 pairs tested), 12 between 2012 and 2013 (48 pairs tested) and 3 between 2013 and 2014 (21 pairs tested). Out of 213 initially seronegative participants, 47 (22.1%) had low antibody titers detected 12 months later (nil-to-low conversions) and 6 (2.8%) had high antibody titers detected 12 months later (nil-to-high conversions), as shown in S2 Table (italicized numbers). The overall seroconversion rate, considering both nil-to-low and nil-to-high conversions, was 24.5 (95% CI, 18.3-32.0) events per 100 person-years of follow-up.”

Finally, we added the following explanation to the legend of Figure 1: “Data from Panel A (see also the italicized numbers in S2 Table) were used to estimate the 12-month seroconversion rate: of 213 initially seronegative participants, 47 (22.1%) had low antibody titers detected 12 months later (nil-to-low conversions) and 6 (2.8%) had high antibody titers detected 12 months later (nil-to-high conversions).”

Reviewer #2: As per my summary and general comments below, the tables and figures need some revision to enhance clarity.

Please see my response below.

Summary and General Comments:

Reviewer #1:

Comments:

In the present study, the authors applied repeated antibody testing to estimate the prevalence of anti-Strongyloides IgG responses and identify incident infections in five farming settlements in the Amazonas State of Brazil. In 2010, the authors performed a longitudinal, serological study in different rural settlements. However, for comparison, only one fecal examination was performed in 2010 in enrolled individuals, without further parasitological or molecular testing.

The manuscript has an adequate title and is written in sound English language.

Major Comments:

-Material and Methods, lines 165ff.: How was the transport chain and continuous freezing guaranteed for the sensitive biological samples from the remote Amazon area? Please comment on shipment and storage of samples. From the description, it is not clear whether the immunoassays were carried out soon after collection, or whether they were carried out only most recently. Please explain.

Thanks for pointing this out. We added further details to the main text regarding sample collection, plasma separation, sample storage, and shipping:

“Venous blood samples were drawn into heparinized vacuum tubes during house-to-house visits carried out from 8 am to 2 pm between 2010 and 2014. Samples were kept on ice packs until centrifugation for plasma separation, within up to 8 hours after collection, in our field laboratory in Acrelândia, Acre, situated 120 km west of the Remansinho area (S1 Fig.). Plasma aliquots were kept in Acrelândia at -20°C for up to four weeks and shipped on dry ice to our research laboratory in São Paulo, where they were stored at -70°C until tested. Samples were screened for anti-*Strongyloides I*gG antibodies in 2022, with a total of 898 plasma samples analyzed.” (Methods, Study design and population, second paragraph).

-Materials and Methods, study population: The authors stated that only patients with microscopically confirmed infections were treated with anti-parasitic drugs. What happened with the serologically positive individuals with antibodies against Strongyloides and especially with those who seroconverted? Is there any federal guideline from the Brazilian Ministry of Health on how to deal with those individuals?

Because serological tests were carried out about 10 years after blood collection, the antibody status of study participants was not available for timely treatment of seroconverters. There is no specific guideline in Brazil regarding ivermectin treatment of apparently healthy people with anti-*Strongyloides* IgG antibodies, even when seroconversion events are identified. We added the following sentence to the main text: “Because antibody tests were carried out approximately 10 years after the field study was concluded, no attempt was made to administer ivermectin to seroconverters in this highly mobile population.” (Results, Seroconversion and seroreversion rates, second paragraph).

-Results, Lines 278-287: Since the presence of other intestinal helminth infections is important for the interpretation of positive serology for strongyloidiasis, the authors should give a brief description of parasitological results here, at least in terms of presence (n; %) of other intestinal helminth infections.

Regarding results of stool examination: please see the absolute numbers and proportions of positive tests according to species in S1 Table. These results are briefly mentioned in the main text:

“Only 14 (9.2%; 95% CI, 5.1-15.0%) of 152 participants tested during survey 1 had helminth eggs or larvae detected by stool examination. Two of them passed *S. stercoralis* larvae (prevalence, 1.3%) and both were positive for specific IgG antibodies (S1 Table). Similar proportions of helminth-positive (7 of 14; 50.0%) and helminth-negative (65 of 138; 47.1%) participants had anti-*Strongyloides* antibodies at the baseline (Fisher exact test, P = 1.000).” (Results, Antibody prevalence and its correlates, second paragraph).

Why parasitological exams were discontinued in the following years?

Obtaining stool samples for microscopic examination proved to be quite challenging in this population. First, there is no electricity in the community; therefore, samples could not be kept at 4°C until the time they were retrieved by our field team. We provided formalin-containing plastic containers for preserving samples, but there are several safety concerns regarding this use. Second, the study area is quite extensive and repeated visits to each household were required to retrieve stool samples (which were rarely available the next day after our first visit).

-Results, Lines 338-340 and 345-348: The authors should not include and discuss speculative comments into the Results section.

Corrected.

Lines 338-340: “Overall, untreated individuals with high IgG antibody levels at the baseline usually remained seropositive over the next years, most likely due to frequent re-exposure to infective S. stercoralis larvae in the soil, chronic carriage of adult female worms, or both.” We deleted the final part of the sentence (starting with “…, most likely due to…”). The deleted comment was placed in the Discussion section: “We suggest that participants with long-lasting high-level antibody responses may be frequently re-exposed to infective *S. stercoralis* larvae in the soil, may chronically carry adult female worms, or both.” (Discussion, 4^th^ paragraph).

Lines 345-348: “Untreated individuals with low-level and transient IgG response to larval antigens may had been exposed to third-stage larvae that failed to develop into adult worms, may have cleared infections with adult S. stercoralis females spontaneously, or may present cross-reactive antibodies with other STHs.” We deleted this sentence from the Results section.

We added the following sentence to the Discussion section: “Low-level and transient IgG responses to larval antigens may be due to exposure to third-stage larvae that failed to develop into adult worms, spontaneous clearance of infections with adult S. stercoralis females, or the presence of cross-reactive antibodies with other STHs.” (Discussion, 4^th^ paragraph).

-Results: My major concern is the absence of parasitological exams in follow up visits and the very speculative discussion on Strongyloides-derived seroconversion during the longitudinal study, without a strong, parasitological fundament (Baermann, APC or PCR). Additional parasitological exams (e.g. K-K or HPJ), even not adequate for the diagnosis of Strongyloides, would, at least, be useful to exclude the presence of other soil-transmitted helminth infections. As stated before by the authors, this might facilitate the participation of other helminth infections and the occurrence of cross-reactivities, which should not be attributed to the presence of Strongyloides. This weak point is critically addressed and discussed by the authors in the manuscript, however, does not reduce my concerns in terms of validity of the results for the epidemiology of strongyloidiasis, especially in this remote Amazon area, where other intestinal infection are supposed to be frequent. A possibility for reducing the cross-reactivity with hookworm or Ascaris-derived antigens would be the preabsorption of plasma samples with these antigens. Did the authors consider this?

We agree that cross-reactive antibodies elicited by infection with other STHs are a reason for concern. However, we note that infection with other intestinal nematodes is relatively infrequent in the study population, as judged by the results of 152 fecal-based tests carried at the baseline (S1 Table). More specifically, only 4 (2.6%) participants had *Ascaris* eggs, 5 (3.3%) had *Trichuris* eggs, and 4 (2.6%) had hookworm eggs detected on a single stool sample by a sedimentation-concentration technique (similar to the original HPJ method).

Reassuringly, “similar proportions of helminth-positive (7 of 14; 50.0%) and helminth-negative (65 of 138; 47.1%) participants had anti-Strongyloides antibodies at the baseline (Fisher exact test, P = 1.000).” (Results, Antibody prevalence and its correlates, second paragraph).

These results may be surprising, given that remote rural populations are usually expected to be heavily exposed to STHs. Nevertheless, our recent population-based surveys across the Amazon have shown relatively low prevalence rates of infection with intestinal helminths in the general population (e.g., doi: 10.1590/s0102-311x2007000200019 [hookworm, 7.7%; *Ascaris,* 5.8%; *Strongyloides,* 2.6%; *Trichuris,* 2.3%; 429 samples examined)] and even among children (doi: 10.1590/s0102-311x2007000600004; the most common STH, *Ascaris,* was detected in only 17 or 3.1% of the 554 under-five children examined). Interestingly, in a study of immune responses to *Mansonella* *ozzardi* filariae, we screened for intestinal helminths all 84 participants aged 10-98 years living in riverine Amazonian communities, by using a standard sedimentation-concentration technique (doi: 10.1371/journal.pntd.0006327). We failed to find a single participant with a current STH infection detected by microscopy!

We added the following comment to the Discussion section:

“We note, however, that carriage of intestinal nematodes is relatively infrequent in the study population, as judged by the results of 152 fecal-based diagnostic tests carried at the baseline (S1 Table), most likely due to the frequent self-administration of anthelminthics.” (Discussion, 4^th^ paragraph).

Given the low prevalence of *Ascaris* infection in our study population, we did not consider pre-adsorbing our plasma samples with *Ascaris* antigens.

-Discussion: The authors might include some discussion on how these seropositive individuals should be dealt with and whether MDA campaigns with ivermection should be considered. This might be important for the scenario of anti-Strongyloides campaigns in Brazil and elsewhere.

Thanks for raising this critical issue. The most recent guideline for strongyloidiasis control, published by the World Health Organization in 2024, suggests that preventive ivermectin treatment (MDA) must be introduced in populations with community-wide prevalence of specific IgG antibodies >25% (or >10% among school-aged children) and/or community-wide fecal-based positivity >10% (or >5% among school-aged children) (reference 28, page xiv).

We added the following sentences to the Discussion section:

“Importantly, community-wide seroprevance rates in our and other Amazonian populations often exceed the 25% threshold to initiate preventive chemotherapy for strongyloidiasis control, according to the 2024 World Health Organization guideline [27,28].” (Discussion, second paragraph).

“We argue that repeated serosurveys can provide key information for planning, monitoring, and evaluating control interventions – e.g., those based on ivermectin preventive chemotherapy where the community-wide prevalence of infection diagnosed by stool examination exceeds 10% or the seroprevalence exceeds 25% [27,28] – across the Amazon and other endemic regions in the tropics.” (Discussion, 5^th^ paragraph).

Minor Points:

-Materials and Methods, Line 214: Please use ‘higher’ instead of ‘greater’.

Corrected.

-Results, Lines 323-324: The Results section should not contain a discussion part and indications of references from other research groups [ref 22 and 23]. This is very speculative here, since no further fecal examinations were conducted.

Thanks for pointing this out. The sentence reads: “No seroreverser had received ivermectin treatment for strongyloidiasis, although many had reported recent deworming with albendazole or mebendazole at doses with little efficacy against *S. stercoralis* [22,23].”

Here, we provide relevant information on treatment that is crucial to interpret seroreversion events (“no seroreverser had received ivermectin treatment for strongyloidiasis”), but emphasize that, in our population, “self-administered deworming with albendazole or mebendazole” is a common practice. To place this information in a proper context, we note that albendazole or mebendazole at the doses commonly used by our study participants have little, if any, effect on *Strongyloides* and cite two references (22 and 23) that support this statement.

We changed the text to: “No seroreverser had received ivermectin treatment for strongyloidiasis during our study, although many reported recent deworming with albendazole or mebendazole. Importantly, these anthelminthics were commonly used at doses with little, if any, efficacy against S. stercoralis [22,23].”

Reviewer #2: This paper addresses an important topic and my overall impression is that it is mostly well-written, with a thorough description of the laboratory procedures. Some clarifications to the manuscript are required to better orient the reader, to improve the clarity in presentation of the results, and to discuss the public health implications of the findings. I suggest the following issues to be addressed:

1. Title – is dynamics really the right word?

We believe so. This is the most commonly used word to describe results from repeated serosurveys. For instance, one of the classical studies on antibody dynamics in malaria, authored by a team of native English speakers (White MT, Griffin JT, Conway DJ, Riley EM, et al.), is entitled “Dynamics of the antibody response to Plasmodium falciparum infection in African children” (J Infect Dis. 2014; 210(7):1115–22; doi: 10.1093/infdis/jiu219).

2. Line 94 [ref. 5; but see also ref 6] – it’s not clear why you’ve chosen to cite the references this way. Please clarify why you’re not just referencing both [5] and [6].

Thanks for pointing this out. Reference 5 is a narrative review of diagnostic techniques that highlight the limitations of fecal-based tests in large-scale population surveys. Reference 6, however, provides a nice yet relatively rare example of a large field study in Ecuador where both fecal- and antibody-based diagnostic methods were used, showing that fecal-based diagnosis can be carried out in the field under specific circumstances.

3. The low sensitivity of faecal-based diagnostics is well described, but can you clarify the issue with diagnostics further. Is the low sensitivity more of a problem clinically, or just for epidemiological surveillance?

Thanks, this is a critical issue. Actually, the low sensitivity of currently available diagnostics affects both the clinical practice and community-wide surveillance. We changed the text to mention this point:

“More sensitive and practical diagnostics for use in resource-poor laboratories in endemic countries are needed to identify and treat cases, eliminate potential sources of infection, and accelerate progress toward the control of strongyloidiasis through population-based surveillance [1,5].“ (Introduction, second paragraph).

4. Line 400. Cross-reactivity with other locally prevalent STHs is mentioned but can you be more specific as this is a vital component to understand in order to interpret your findings? Either expand on this here, or perhaps it is better suited in the discussion.

Please see our responses on this topic to reviewer #1: “We agree that cross-reactive antibodies elicited by infection with other STHs are a reason for concern. However, we note that infection with other intestinal nematodes is relatively infrequent in the study population, as judged by the results of 152 fecal-based tests carried at the baseline (S1 Table). More specifically, only 4 (2.6%) participants had *Ascaris* eggs, 5 (3.3%) had *Trichuris* eggs, and 4 (2.6%) had hookworm eggs detected on a single stool sample by a sedimentation-concentration technique (similar to the original HPJ method). Reassuringly, “similar proportions of helminth-positive (7 of 14; 50.0%) and helminth-negative (65 of 138; 47.1%) participants had anti-Strongyloides antibodies at the baseline (Fisher exact test, P = 1.000).” (Results, Antibody prevalence and its correlates, second paragraph).”

We added the following sentence to the Discussion section: “Low-level and transient IgG responses to larval antigens may be due to exposure to third-stage larvae that failed to develop into adult worms, spontaneous clearance of infections with adult S. stercoralis females, or the presence of cross-reactive antibodies with other STHs. We note, however, that carriage of intestinal nematodes is relatively infrequent in the study population, as judged by the results of 152 fecal-based tests carried at the baseline (S1 Table), most likely due to the frequent self-administration of anthelminthics.” (Discussion, 4^th^ paragraph).

5. Line 116. One-fourth – change to a quarter or 25%

Corrected to 25%, thanks.

6. Line 137 onwards. It’s very helpful for readers to have more background information about the setting, e.g. healthcare access, broader health status. Why were these communities chosen and how representative are they – are the results applicable to other populations?

Thanks for raising this point. We added additional information regarding the study site. The revised text (Methods, Study Site) now reads:

“We measured anti-Strongyloides IgG antibodies in remote populations from southern Amazonas state, Western Brazilian Amazon, next to the border with Bolivia (S1 Fig.). The study site is characterized by an equatorial humid climate (annual average temperature, 26.4C), with most rainfall between November and March (annual average, 2,318 mm). There are five small farming settlements in the area, where the main economic activities are subsistence agriculture and logging in the nearby rainforest [17]. Newly opened informal farming settlements such as our study site, characterized by poor housing and lack of basic infrastructure, are commonly seen across the “deforestation arc” of the Amazon, especially along or next to major roads such as the BR-364 and BR-319 interstate highways. Tropical diseases such as malaria and STH infections are expected to be highly prevalent in these settings.

The main settlement, Remansinho, is situated along the final 40 km of a 60 km-long unpaved road originating from the BR-364 interstate highway, known as Ramal do Remansinho, while the other four are situated along secondary roads known as Ramal da Linha 1, Ramal da Castanheira, Ramal dos Seringueiros, and Ramal dos Goianos, all originating from the main unpaved road. Ramal da Linha 1 and Ramal da Castanheira are older settlements, opened in the late 1990s, whereas the colonization of the other areas started only in 2007. There is no electricity or piped water supply in the area. Health care access is very limited in the Remansinho area. There is no formal health facility in the community, except for a malaria diagnosis post with a microscopist. Patients seek basic health care in the nearest village, Nova Califórnia (40-60 km away by a dirt road), and more specialized care in a regional hospital in the village of Extrema, >80 km away.”

7. Ethics statement. This is a thorough description (probably not all needed for the manuscript), however doesn’t mention the purpose of the original study. Am I correct in thinking the samples were originally taken (and participants consented) to a study about malaria? Did participants consent for future research but not explicitly for a study about strongyloidiasis?

Please see the response reproduced from page 2:

“At the time this project was evaluated by the Institutional Review Board of the University Hospital of the University of São Paulo and subsequently by the National Human Research Ethics Committee of the Ministry of Health of Brazil, in early 2010, there was no National legislation addressing the establishment of biological collections resulting from field-based surveys. Although the original research project focused on acute febrile illnesses, such as malaria and dengue, the research plan explicitly included further screening for other health conditions that were judged clinically or epidemiologically relevant by the field physicians and nurses. Under this criterion, additional testing was done, at discretion of the field team or by request of study participants, for cutaneous leishmaniasis (by microscopy), Mayaro fever (by RT-PCR), intestinal parasites (by stool examination, as described in the present manuscript), and anemia (hemoglobin measurement). We detected anti-*Strongyloides* antibodies on plasma samples from study participants stored in our biological collection at the Institute of Biomedical Sciences, University of São Paulo, because a high seroprevalence of strongyloidiasis has recently been described in a similar Amazonian setting (doi: 10.1590/S1678-9946202466072).

We added the following sentence to the “Ethics statement: “Plasma samples tested for anti-*Strongyloides* antibodies were part of the biological collection, maintained at the Institute of Biomedical Sciences, University of São Paulo, that resulted from 10 consecutive cross-sectional surveys carried out in the study site.”

At the time of the house-to-house surveys, study participants agreed to provide blood samples for malaria-related tests and other health conditions that were judged clinically or epidemiologically relevant by the field physicians and nurses, but strongyloidiasis was not explicitly mentioned in our consent forms.”

8. Line 166. If normally distribute please present mean and standard deviation. If not, median and interquartile range.

Corrected. Age is normally distributed. We provide the standard deviation.

9. Line 168. ‘Because of the high mobility of the local population, only 5.4% (n=24) of the study participants had specific antibodies measure in study years’. I don’t understand what you mean here. Please clarify.

This is a panel survey, with repeated measurements on the same subjects. When the study population is very mobile, finding the study subjects during consecutive house-to-house surveys over 5 years is particularly challenging.

We changed the text in order to make this point clearer: “Because of the high mobility of the local population, only 5.4% (n = 23) of the study participants were present in the site and had specific antibodies measured in all study years; 180 (42.2%) were tested for antibodies only once, 116 (27.2%) were tested twice, 57 (13.4%) were tested three times, and 50 (11.7%) were tested four times.”

10. It’s not very clear from the methods how many participants had which type of samples and tests, and the sequencing of this. Perhaps a table in the results section – with rows depicting samples and test and columns dates of surveys, would be helpful to orientate the reader.

The reviewer is absolutely right in pointing that the study population was poorly described. The main study focuses on antibody prevalence data from 898 plasma samples obtained from 426 participants (1 to 5 samples per participant). The number of samples tested each year is shown in Table 1 and ranges between 244 (in 2010) and 101 (in 2014).

We changed the text to:

“A total of 584 residents were enumerated during the consecutive cross-sectional surveys (1 through 10) between 2010 and 2014. Sociodemographic and morbidity information and venous blood samples were collected from consenting residents [17]. (…) The study population comprises 426 study participants ranging from 4 months to 79 years of age (mean, 35.9 years; standard deviation, 0.61), distributed into 160 households, who had one or more plasma samples available for antibody testing (72.9% of those enumerated during our census surveys).”

Regarding the number of samples tested for antibodies per participant, we state the following:

“Because of the high mobility of the local population, only 5.4% (n = 23) of the study participants were present in the site and had specific antibodies measured in all study years; 180 (42.2%) were tested for antibodies only once, 116 (27.2%) were tested twice, 57 (13.4%) were tested three times, and 50 (11.7%) were tested four times.” (Methods, Study design and population, second paragraph).

We also did some analyses on study participants who were tested for intestinal parasites by stool microscopy. This subpopulation (n = 152) is described as follows:

“During survey 1, participants were given plastic containers containing 10% formalin (Coprotest, NL, Campinal, Brazil) and asked to provide one stool sample for detection of eggs, cysts, and larvae of intestinal parasites. We used a simple sedimentation-concentration technique in 100-mm conic-bottom test tubes [19,20] that appears to be nearly as sensitive for the diagnosis of strongyloidiasis as the Baermann and agar plate culture methods [20]. Overall, 152 participants who were tested for anti-Strongyloides antibodies in survey 1 had also stool samples collected and examined for intestinal parasites.” (Methods, Study design and population, third paragraph).

11. Please clarify – was it just participants with faecal diagnosis of strongyloidiasis that were offered treatment with ivermectin? Not those with positive serology?

Only stool-positive individuals (n = 2) were treated. We clarify this point as follows:

“Because antibody tests were carried out approximately 10 years after the field study was concluded, no attempt was made to administer ivermectin to seroconverters in this highly mobile population.” (Results, Seroconversion and seroreversion rates, second paragraph).

12. Line 209 – ‘with confirmed S. stercoralis infection’ – how was it confirmed? Please make it clear throughout what you took to be the gold-standard confirmation of diagnosis.

Information added: “All microplates included positive controls (a pool of antibody-positive plasma samples from patients with S. stercoralis infection confirmed by the Baermann concentration method)…”

13. Line 227 onwards. Description of variables in logistic regression model well described, but how was the model built? Did you put all the variables in together and only keep the statistically significant ones, or did you sequentially add them one by one. If the latter, what order did you add them?

Thanks for raising this point.

We first run unadjusted models in which each of the variables listed below were tested separately (see Table 1, left-hand colums):

“Unadjusted models comprised the following individual-level covariates: age (stratified as ≤ 5, 6-15, 16-30, 31-50, and >50 years); gender; reported treatment with locally available anthelminthics (albendazole 400 mg, single dose; or mebendazole, 100 mg twice a day for 3 days) within the past 6 months (yes/no); having passed helminths in the feces within the past 6 months, either spontaneously or following anthelminthic treatment (yes/no); and type of stool disposal (pit latrine vs. open defecation). Household-level covariates were wealth index quartile; presence of a groundwater well in the property (yes/no); housing material (bricks, wood, or other materials, such as adobe or palm leaves); and place of residence (farming settlement), in addition to survey year (2010, 2011, 2012, 2013 or 2014).”

We next built a multivariable model comprising all variables associated with P < 0.20 in initial unadjusted analyses (i.e., age, gender, type of stool disposal wealth index, place of residence (farming settlement) and survey year). These are listed in the right-hand columns of Table 1. These variables “were entered in multivariable regression models built for all surveys combined; participants with missing information (n = 44) were excluded from the final model.”.

14. Line 253 – how were confidence intervals estimated?

We used the exact formulae to estimate 95% confidence intervals, which are based on standard Fisher's exact test, given by Rothman KJ, Boice JD Jr: Epidemiologic analysis with a programmable calculator. NIH Pub No. 79-1649. Bethesda, MD: National Institutes of Health, 1979;31-32. The procedure is explained, and the formulae are given at https://www.openepi.com/PDFDocs/PersonTime1Doc.pdf, page 3. The reader can reproduce these results by entering the number of cases (n = 53 seroconversion events) and the number of person-years of follow-up (216.3) into open-access web-based calculators such as OpenEpi (https://www.openepi.com/PersonTime1/PersonTime1.htm).

15. Results section – as above I think a table denoting what samples and tests were done (with timings) would be helpful at the beginning of the results section.

Please see our response above. We provided further details, in the Methods section, regarding the study population tested for antibodies. Furthermore, Table 1 provides the numbers of samples tested per year. We believe that this information (in the revised manuscript) is clear enough for the reader.

16. Line 263 – can you present how many individuals ever had positive serology first, then how many samples in total?

I am not entirely sure whether I understood your question. Please notice that our denominator is the number of samples tested (n = 898), instead of the number of individuals (n = 426). This is because up to five samples were tested per individual. Indeed, we first provide the number (n = 465) of positive samples, followed by the number of samples tested (n = 898) and by the proportion (51.8%) with 95% confidence intervals (48.4-55.1%): “Overall, 465 of 898 (51.8%; 95% CI, 48.4-55.1%) plasma samples collected from 426 study participants over four years were positive (…)”

The number of samples tested per individual is variable (ranging between 1 and 5; please see Methods, Study design and population, second paragraph and my response on page 11). Therefore, estimating the number of participants who had at least one positive test is meaningless in this context. For example, 180 (42.2%) participants had a single sample tested, while 23 (5.4%) had consecutive 5 samples tested. People tested multiple times are much more likely to have at least one positive sample.

17. Table 1 needs some clarification. The n/N(%) column needs a clearer explanation e.g. Positive S. stercoralis ELISA/number of samples tested (seroprevalence, %). I don’t think it’s enough to only have the explanation in the ledger.

We improved the description in the footnote as follows:

“n = number of positive samples with IgG antibodies detected by ELISA among participants within the exposure category.

N = total number of samples from participants within the exposure category that were tested for IgG antibodies by ELISA.

Percentages (n/N × 100) correspond to the seroprevalence within each exposure category.”

The overall seroprevalence is described in the main text: “Overall, 465 of 898 (51.8%; 95% CI, 48.4-55.1%) plasma samples collected from 426 study participants over nearly five years were positive.”

18. Table 1. Spelling of antihelminthics needs correcting in table 1.

Both “anthelminthics” and “antihelminthics” are used in English (please see, for example, https://www.ncbi.nlm.nih.gov/books/NBK548602/). Note that “anthelminthics” is the most common form, although “antihelminthics” is perfectly correct (although the World spelling corrector insists to highlight this form…).

19. Table 1. Use of antihelminthics and passed helminths – it needs to be clear that this is self-reported from a survey (if that’s correct).

Yes, this information is self-reported. This information was added to Table 1. Note that these variables were also described in Methods, Statistical analysis: “reported treatment with locally available anthelminthics (albendazole 400 mg, single dose; or mebendazole, 100 mg twice a day for 3 days) within the past 6 months (yes/no); reported having passed helminths in the feces within the past 6 months, either spontaneously or following anthelminthic treatment (yes/no)”.

20. Line 273 – although increase in antibody prevalence noted, the confidence intervals overlap.

“Antibody prevalence rates increased over the study period and ranged from 45.9% (95% CI,

274 39.5-52.4%) in 2010 to 61.1% (95% CI, 51.4-70.1%) in 2013. Indeed, survey year was the only covariate found to be significantly associated with seropositivity in multivariable logistic regression analysis (P-value for trend = 0.009; Table 1).” Therefore, although CIs overlap, the adjusted multivariable analysis indicates that there is a significant trend towards increased seroprevalence with survey year.

21. Line 281 – ‘periodic deworming was a common practice’. Can you be more specific about this (expanding on it in the discussion section would be helpful).

This point refers to the self-reported use of anthelminthics within the past six months (please see our response on page 8):

“Periodic deworming was a common practice and a likely reason for the low prevalence of STH infection observed in the population. Nearly a quarter of study participants – a total of 200 (24.3%) out of 822 observations; information missing for 76 – reported having taken albendazole or mebendazole within the past six months, regardless of any symptom or laboratory diagnosis of infection.” (Results, Antibody prevalence and its correlates, second paragraph).

This means that 24.3% of the study participants reported recent use of anthelminthics – hence this practice is described as “common” in the study population. This is far from surprising: anthelminthics are cheap and widely available across rural communities where we have carried out prevalence surveys over the past decades. They are routinely prescribed to people in all age groups even in the absence of clinical signs and symptoms suggestive of infection and can be easily purchased in local drugstores.

22. Line 282 – one fourth – change to a quarter or give exact percentage.

Corrected to “a quarter”.

23. Figure 1. This is helpful, but is it missing the ledger to explain what A, B, C & D refer to?

Thanks for point this.

The required information regarding each figure panel (from A to D) is provided in the Figure 1 legend: “Panel A, blood draws approximately 12 months apart (data from 2010 vs. 2011, 2011 vs. 2012, 2012 vs 2013, and 2013 vs. 2014; n = 408 comparisons); Panel B, blood draws approximately 24 months apart (data from 2010 vs. 2012, 2011 vs. 2013, and 2012 vs. 2014; n = 262 comparisons); Panel C, blood draws approximately 36 months apart (data from 2010 vs. 2013 and 2011 vs. 2014; n = 130 comparisons); Panel D, blood draws approximately 48 months apart (2010 vs. 2014; n = 45 comparisons).”

We also added the following sentence to the legend to make it clear how 12-month seroconversion rates were calculated:

“Data from Panel A (see also the italicized numbers in S2 Table) were used to estimate the 12-month seroconversion rate: of 213 initially seronegative participants, 47 (22.1%) had low antibody titers detected 12 months later (nil-to-low conversions) and 6 (2.8%) had high antibody titers detected 12 months later (nil-to-high conversions).”

24. Figure 2. Again needs a ledger to explain the colour grading. All tables and figures should be revised to make sense alone without having to refer to the results section text for interpretation.

Please refer to Figure 1 legend to properly interpret the color code in our standard Sankey diagram that depicts the flow from baseline test results (stratified as “negative”, “low” and “high”) to test results in the same people 12 months later (again stratified as “negative”, “low” and “high”):

“Anti-Strongyloides IgG responses were stratified as negative (absorbance < 0.286; **light gray**), low (absorbance between 0.287 and 0.561; **light purple**) and high (absorbance > 0.561; **purple**), with the absorbance value of 0.561 corresponding to the median absorbance value among positive samples during the study.”

25. Line 323 – exactly how many had reported recent deworming with albendazole or mebendazole? This is important as could potentially explain seroreversion.

We fully agree that anthelminthic use is a potential confounder. Please see above: 24.3% of study participants reported recent use of anthelminthics (main text and Table 1). However, as discussed above (response to reviewer #1), we emphasize that “anthelminthics (albendazole or mebendazole) were commonly used at doses with little, if any, efficacy against S. stercoralis [22,23]” (Results, Seroconversion and seroreversion rates, 4^th^ paragraph).

Regarding albendazole, please refer to the main conclusion of the study described in reference 29: “The mass administration of albendazole appears to have a negligible impact on the local prevalence of strongyloidiasis”.

The routine use of albendazole and mebendazole is unlikely to have affected seroprevalence rates, but certainly had an impact on the prevalence of other STHs that are sensitive to these anthelminthics.

26. Line 332 – please correct the typo. Samples -> sample.

Thanks, corrected.

27. Discussion section: Can you add a very brief summary of the key results at the beginning of the discussion? I think it would be clearer if you started the discussion with the paragraph beginning line 371 (and paragraph line 377) – then put these into context with the prevalence estimates from the literature (line 361).

Done as suggested.

28. Limitations – the samples were collected over ten years ago, this should be discussed.

Thanks for raising this point, which had also been mentioned on page 3. We changed the main text to:

“An additional limitation is the use of plasma samples collected over one decade ago. Because these samples have been stored at -70 degrees C before testing, a substantial decrease in antibody concentrations since collection is not expected. However, this study describes antibody prevalence rates between 2010 and 2014, with no further follow-up. Further studies are needed to explore the current clinical and public health significance of anti-*Strongyloides* antibody carriage and how antibody status varies over time in this and other endemic settings and in response to mass treatment, and to identify serological correlates of chronic infection.” (Discussion, last but one paragraph).

29. Conclusion – Do you really mean a diagnostic method for clinical purposes (i.e. treating on the basis of positive serology)? Or do you recommend the method more for epidemiological surveillance and to aid decision making on control programmes?

Please see my response above (page 4). We (and others) suggest the use of serology only for epidemiological surveillance within control programs. The (revised) last paragraph of Discussion reads:

“In conclusion, our results further support the use of serology as a diagnostic method that can be implemented in the field, especially as a tool for monitoring temporal changes in *S. stercoralis* transmission intensity in remote populations and estimating the effectiveness of control interventions at a population level.”
